# Model of gene expression in extreme cold - reference transcriptome for the high-Antarctic cryopelagic notothenioid fish *Pagothenia borchgrevinki*

**DOI:** 10.1186/1471-2164-14-634

**Published:** 2013-09-21

**Authors:** Kevin T Bilyk, C-H Christina Cheng

**Affiliations:** 1 Department of Animal Biology, School of Integrative Biology, University of Illinois at Urbana-Champaign, 515 Morrill Hall, 505 South Goodwin Ave., Urbana, IL 61801, USA; 2 Current Address: College of Fisheries and Life Science, Shanghai Ocean University, 999 Hucenghuan Rd, Shanghai 201306, China

**Keywords:** Notothenioidei, Antarctic fish, Transcriptome, Heat tolerance, Cold adaptation, Cold specialization

## Abstract

**Background:**

Among the cold-adapted Antarctic notothenioid fishes, the high-latitude bald notothen *Pagothenia borchgrevinki* is particularly notable as the sole cryopelagic species, exploiting the coldest and iciest waters of the Southern Ocean. Because *P. borchgrevinki* is a frequent model for investigating notothenioid cold-adaptation and specialization, it is imperative that “omic” tools be developed for this species. In the absence of a sequenced genome, a well annotated reference transcriptome of the bald notothen will serve as a model of gene expression in the coldest and harshest of all polar marine environments, useful for future comparative studies of cold adaptation and thermal responses in polar teleosts and ectotherms.

**Results:**

We sequenced and annotated a reference transcriptome for *P. borchgrevinki*, with added attention to capturing the transcriptional responses to acute and chronic heat exposures. We sequenced by Roche 454 a normalized cDNA library constructed from pooled mRNA encompassing multiple tissues taken from environmental, warm acclimating, and acute heat stressed specimens. The resulting reads were assembled into 42,620 contigs, 17,951 of which could be annotated. We utilized this annotated portion of the reference transcriptome to map short Illumina reads sequenced from the gill and liver of environmental specimens, and also compared the gene expression profiles of these two tissue transcriptomes with those from the temperate model fish *Danio rerio.* From this, we identified a conserved group of 58 GO terms, in which terms related to transcription and its regulation, ubiquitin-protein ligase activity, protein ubiquitination, and protein binding among others are more prevalent in the bald notothen, suggesting the pertinent genes play essential roles in cold temperature functioning.

**Conclusion:**

We sequenced multiple tissue transcriptomes from native and heat-exposed experimental specimens of the high Antarctic, cryopelagic notothenioid *P. borchgrevinki* to construct a reference transcriptome. In a proof of concept, we utilized the annotated reference transcriptome to profile the gene expression patterns of gill and liver, and identified a suite of over and under-represented GO terms when compared to the tropical water zebrafish suggesting these functions may be important for surviving in freezing waters. The transcriptome resource from this study will aid future investigations of cold adaptation and thermal response of polar ectothermic species.

## Background

Modern Antarctic waters are dominated by five highly endemic families of fishes that belong to the perciform suborder Notothenioidei [1]. The evolution of these species in the freezing Southern Ocean waters has led to many shared adaptations to their ice-laden environment. These include newly minted genes with novel functions such as the blood borne antifreeze glycoproteins that prevent ice expansion in their body fluids and preserve life [2], as well as adaptive modifications of pre-existing genes to effectively allow optimal function at low temperatures [3-5]. The evolutionary history of these species in the world’s coldest marine waters makes them excellent models for functional and molecular studies of evolutionary adaptations to extreme environment, however, genomic or transcriptomic level resources for the Antarctic notothenioids, while emerging, currently exist for only a few species [6,7], and are entirely missing for species endemic to the highest latitude and harshest Antarctic marine environment.

While whole genome sequencing remains out of reach for many non-model species, transcriptome sequencing has become more accessible, opening new avenues into genomic level inquiries. To increase the inclusiveness of the diversity of expressed genes, the source RNA can be pooled from many tissues and experimental treatments, providing access to a wider segment of an organism’s genic content and transcriptional repertoire. Prior to sequencing, the transcript pool can also be normalized in order to evenly capture highly expressed genes and the rare transcripts, maximizing transcript representation and diversity. The sequence information from comprehensive transcriptomes has important utilities as it serves as the essential source material for comparing many candidate genes across taxa to help gain insights into the molecular differences that may underlie adaptive changes [8]. Additionally, a well annotated reference transcriptome can be used for many downstream analyses, including RNA-seq approaches to tissue- or genome-wide gene expression profiling, where it serves as a map for quantifying transcript abundance from short sequencing reads [9].

Two previous studies have reported sequenced transcriptomes from Antarctic notothenioids. The first by Chen *et al*. [6] used traditional Sanger sequencing to sequence non-normalized EST libraries from the brain, liver, ovary, and head kidney of a nototheniid, the Antarctic toothfish *Dissostichus mawsoni*. This was aimed at studying transcriptional differences between cold-adapted polar fish and temperate or tropical species. The sequenced *D. mawsoni* tissues shared high levels of expression in a number of genes including several involved in defending the cell from environmental stressors, while other highly expressed genes are tissue specific. When compared to the transcriptional profiles of temperate and tropical model fishes, a subset of the highly expressed genes is specific to the Antarctic toothfish suggesting some role in its cold adaptation. A Gene Ontology analysis of these identified that the dominant transcripts were notably from genes with functional roles as molecular chaperones, in the ubiquitination pathway and in catabolism, as well as including several extracellular proteases.

More recently, Shin *et al*. [7] made a qualitative comparison of transcriptome sequences obtained by Roche 454 sequencing of normalized libraries from three notothenioids, *Notothenia coriiceps*, *Chaenocephalus aceratus*, and *Pleuragramma antarcticum*. The study focused on species differences in the genes present rather than the level of their expression. Comparisons of brain and liver transcriptomes from the three notothenioids to temperate model fishes identified very few functional differences. The most notable among these was the enriched representation of ligase activity, with the authors calling particular attention to members of the ubiquitin-proteasome pathway. This suggests that there are more copies of genes in this pathway in Antarctic notothenioids, consistent with their augmented transcript abundance observed by Chen *et al*. [6] in *D. mawsoni*, and the high levels of ubiquitinated proteins found in high-latitude notothenioid species [10].In addition to this and the Chen *et al*. [6] study, Buckley and Somero [11] and Clark et al. [12] both used cDNA microarrays to investigate changing gene expression in notothenioid fishes with acute exposure to 4°C providing a wealth of additional expression profiles for *Trematomus bernacchii* and *Harpagifer antarcticus* respectively.

Though transcriptome sequences for four notothenioid species are now available, these do not include any of the high-latitude coastal species that exploits the coldest and iciest waters of the Southern Ocean. As frequent experimental species in studies by Antarctic researchers, genomic resources for these high-latitude species remain a major and important need. They are also useful for studies comparing gene expressions in the coldest habitat to those in the relatively milder lower latitudes. Our interest is in one such species, the cryopelagic nototheniid *Pagothenia borchgrevinki* (bald notothen). *P. borchgrevinki* is the only notothenioid that preferentially dwells within the crevices of the voluminous platelet ice layer beneath the surface fast ice of McMurdo Sound [13], the southern limit of marine life and arguably the coldest (chronically near or at −1.9°C, the freezing point of seawater) and iciest extreme of all Antarctic fish habitats. Despite being highly cold-adapted and stenothermal, interestingly the bald notothen retains some degree of thermal plasticity. It is capable of greater tolerance to acute heat challenge if preceded by warm acclimation [14,15], which may have bearing on the species’ fate in face of global climate change.

The goal of this project was to sequence tissue transcriptomes of *P. borchgrevinki*, and develop a reference transcriptome as a model of notothenioid gene expression in the coldest extreme of Antarctic marine environments. The reference transcriptome will allow better understanding of the modifications in this highly cold-adapted cryopelagic species, as well as enable further genomic level studies of other polar teleosts and ectotherms. In addition, given the emergent but still limited understanding of overall diminished heat tolerance in Antarctic notothenioid fishes but varying degrees of continued thermal plasticity, this reference transcriptome was constructed with particular interest and utility for studies of notothenioid response to heat stress, both chronic and acute, by including tissue transcripts from such experimental heating regimes. Also, in conjunction with this reference transcriptome, we used short transcript reads generated by Illumina sequencing to examine the gene profiles of liver and gill, allowing proof of concept investigations into the transcriptional differences between Antarctic fish and tropical water model fish.

## Methods

### Collection of specimens, warm acclimation, acute heat stress, and tissue sampling

All animal care and use were performed following institutional protocol approved by the University of Illinois. Specimens of the Antarctic nototheniid *P. borchgrevinki* were collected by hook and line through the sea ice of McMurdo Sound (77.6°S, 166.8°E) during the austral spring and summer. These were transported back to McMurdo Station in aerated coolers and held for several weeks in the old aquarium located near the Station seawater intake, which received a constant flow of cold ambient seawater that remained below −1.4°C. After this recovery period, 15 *P. borchgrevinki* were transferred into a warm acclimation tank maintained at 4 ± 0.05°C using a submerged 1000 W heater and a temperature controller activated solenoid valve that regulated the addition of cold ambient seawater as described in Bilyk and De Vries [14].

After two, four, and seven days of 4°C warm acclimation, five specimens were sacrificed by severing their spinal column with a scalpel. Brain, liver, spleen, head kidney, white muscle, and gills were dissected from each, and immersed in 10 volumes −20°C sterile 90% ethanol. The tissue samples were then stored at −20°C with the ethanol replaced three times over the first 24 hours. This series of ethanol dehydrations effectively preserve the integrity of DNA and RNA in our samples, as we have extensively tested this method previously and obtained fully intact nucleic acids.

Tissues were similarly collected from five control specimens taken directly from the flow through ambient seawater tank, and from five experimental specimens exposed to acute heat stress. Fish from this latter group were dissected immediately after heating them at 0.3°C/min to their Critical Thermal Maximum (CTMax), which is the temperature at which persistent loss of equilibrium was observed [14]. Additional tissue samples - brain, liver, spleen, and head kidney from specimens acclimated at 4°C for two months, as well as lens samples of ambient water fish were retrieved from our liquid nitrogen frozen tissue bank and included in the tissue set for transcriptome sequencing.

### RNA isolation, transcriptome sequencing, and sequence assembly

The list of tissues from environmental and experimental fish used for generating the reference transcriptome is shown in Table 1. Obtaining a pool of RNA from this wide selection of tissues was aimed at providing a comprehensive collection of the expressed genes across these conditions.

**Table 1:** **Tissue and temperature combinations used in constructing the reference transcriptome of *P. borchgrevinki***

**Treatment**	**Liver**	**Brain**	**White muscle**	**Spleen**	**Head kidney**	**Gill**	**Lens**
**Native**							
**Control, -1 to −1.9°C**	yes	yes	yes	yes	yes	yes	yes
**Warm Acclimation**							
**2 days, 4°C**	yes	yes	yes	-	yes	yes	-
**4 days, 4°C**	yes	yes	yes	yes	yes	yes	-
**7 days, 4°C**	yes	yes	yes	yes	-	yes	-
**2 months, 4°C**	yes	yes	-	yes	yes	-	-
**Acute Heat Stress**							
**Post-CTMax**	yes	yes	yes	yes	yes	-	-

Overview of the tissues included in the reference transcriptome. Total RNA was extracted from one specimen for each tissue/treatment combination and pooled at equal concentrations. *P. borchgrevinki* reference transcriptome was then produced by sequencing this pool on a Roche 454 GS FLX+ Titanium sequencer.

Total RNA was extracted from 100-200 mg of each tissue sample using the Ultaspec-II RNA isolation system (Biotecx Laboratories Inc., Houston, TX) according to the manufacturer’s instructions. The final RNA pellet was dried and dissolved in in 50–150 μL of 0.5x Tris-EDTA buffer (TE) (5 mM Tris–HCl, 0.5 mM EDTA, pH 8.0), and then treated with 2 units of DNase I (New England Biolabs Inc., Ipswich, MA) at 37°C for 10 minutes to degrade potential contaminating genomic DNA. The DNase I treated RNA was purified using E.Z.N.A. HiBind Mini Columns (Omega bio-tek Inc., Norcross, GA). The concentration and purity of the RNA eluted from the spin columns were determined using a NanoDrop spectrophotometer (ND-1000, Thermo Scientific Inc., Waltham, MA) and its integrity confirmed by running 2 μg of each sample on a 1% denaturing formaldehyde agarose gel.

A pool of RNA was created by mixing one μg from each purified tissue RNA sample. This pool was precipitated with ethanol and the precipitated RNA was redissolved in a final volume of 60μL of TE. The concentration, purity, and integrity were confirmed as described above. The concentrated, pooled RNA sample was submitted to the University of Illinois Roy J. Carver Biotechnology Center, where it was used to generate a normalized cDNA library. This library was sequenced on one full Pico Titer Plate on a Roche454 GS FLX+ Titanium sequencer. The resulting short reads were loaded into gsAssembler v. 2.6 (Roche 454 Inc., Branford, CT) and assembled with the program’s cDNA assembler, using the parameters of a minimum overlap of 60 nt (nucleotide), and minimum shared identity of 97%. Reads were screened against the UniVec core database (downloaded 9/20/2011) to remove common contaminant and vector sequences. Finally, a Biopython script was used to remove any assembled contigs shorter than 100 nt in length. The raw reads used in constructing this transcriptome have been deposited in NCBI’s Sequence Read Archive under Accession number SRP018876.

### Clustering and annotation of the reference transcriptome

Since multiple contigs could result from the same gene owing to either alternative splicing or from errors during sequencing and assembly, we attempted to remove the redundancy that arose from errors by clustering the sequence contigs. This consisted of first performing a self-BLAST using an e-value cutoff of 10^-9^, followed by a Biopython script that grouped sequences sharing at least 95% nt identity and 70% contig length. If multiple contigs belonged to a cluster, only the longest was retained in the reference transcriptome. We did not perform more aggressive clustering to avoid removing biologically relevant variation from alternative splicing or closely related members of gene families.

The contigs that remained after clustering were then screened against public databases to assign putative gene identities. Database screening was done in a tiered fashion, starting with the most specific and well annotated database, then moving to more general sources for contigs that produced no matches. Using BLASTx we screened SwissProt (downloaded 6/2012), UniProt/TrEMBL (downloaded 6/2012), and NCBI’s NR (Non-Redundant) protein database in that order (downloaded 2/2012). If no matches were found in these three databases the contigs were checked against NCBI’s NT (Nucleotide; downloaded 2/2012) database using BLASTn. Both BLASTx and BLASTn searches were performed with an e-value cutoff of 10^-9^ to control the false discovery rate from the multiple comparisons.

Contigs with hits in either SwissProt or UniProt/TrEMBL formed the annotated transcriptome. Due to the less descriptive information available for the contigs with matches only in the NR and NT databases, these were only used in estimating the total number of expressed genes but were not used in the later analysis. Gene Ontology (GO) assignments were made from the UniProt Gene Ontology Annotation file downloaded from EMBL-EBI on 6/2012 based on the accession number of each contig’s highest scoring BLAST hit.

### Comparing the *P. borchgrevinki* reference transcriptome to ***Danio rerio*** (zebrafish) transcriptome

The GO terms associated with the *P. borchgrevinki* reference transcriptome were processed using the GO slimmer available from the AmiGO website with their generic rule set [16]. The resulting set of GO-slims is a subset of the original terms, providing a broad overview of the functional roles of the studied genes. This slimmed GO set was used here to make high-level comparisons with other teleost transcriptome. Despite their great phylogenetic distance, we choose to perform this comparison against the zebrafish, *Danio rerio*, downloaded from Ensmbl on 6/15/2012 [17] as the transcriptome of this well-studied model species is presumably near completely delineated.

To remove differences in annotation as a confounding factor in the comparison, we annotated the zebrafish transcriptome as described for our *P. borchgrevinki* reference transcriptome. A chi-square test of independence was then used to compare the incidence of GO-slim terms between transcriptomes with p-values, and corrected for multiple testing using Benjamini and Hochberg's method [18]. GO-slim terms with a corrected p-value of 0.01 or lower were then considered to significantly differ.

### Illumina sequencing of liver and gill transcripts

To measure transcript abundance and identify tissue specific expression we sequenced liver and gill samples from three specimens taken from ambient water temperatures. Liver was selected for further investigation as it is the most metabolically active internal organ in vertebrate animals and the site of synthesis for most plasma proteins. Differences in gene expression compared to warmer water fishes can be important towards understanding organismal adaptations in *P. borchgrevinki* cryopelagic life history. Gill was selected as a complement, as it is a surface tissue directly confronted by the severe ice-laden marine environment, it is highly metabolically active, being the major site of osmoregulation and respiration, and also because it has largely been ignored in prior investigations of notothenioid transcriptomes. Furthermore, both tissues are frequent targets for studies of heat tolerance, making baseline measurements of gene expression helpful for putting the results of such studies into biological context.

A barcoded cDNA library was constructed for each liver and gill RNA sample using the TruSeq RNA sample prep kit (Illumina Inc. San Diego, CA) and quantified at the University of Illinois Roy J. Carver Biotechnology Center. The six libraries were sequenced on two lanes on an Illumina HiSeq 2000 sequencer, and analyzed with Casava1.8 (pipeline 1.9), producing a collection of 100 nt single-end reads.

These reads have been deposited in NCBI’s Sequence Read Archive along with the 454 reads used to construct the transcriptome under Accession number SRP018876. The Illumina reads for each specimen were screened using the FASTX-Toolkit [19] to remove the first 15 and last 6 nts. Additionally, reads were discarded if they contained any runs of “N”, if 90% or more of the sequence had a Sanger quality score below 23, or if they contained all or part of the 64 nt adaptor sequence. After processing, the program FastQC [20] was used to confirm the quality of all the “cleaned” libraries of reads. To count the number of sequence reads derived from a given transcript by library, the reads were mapped against the annotated transcriptome using the program Bowtie v. 0.12.7 [21]. Bowtie was run using the “best” criterion, set to allow mappings up to a total quality score difference of 70, and to discard any ambiguous reads that mapped to multiple locations in the reference transcriptome. Counts mapped to a given transcript in the annotated transcriptome were enumerated by library using a Biopython script with the pysam python library.

### Isolation and comparison of liver and gill gene expression profiles

The read counts resulting from Illumina sequencing were screened to isolate the expression profile of each tissue. To prepare these profiles for comparison, the counts of individual transcripts had to be normalized for differences in total library size. Normalization was necessary so that differences in sequencing depth would not be confounded with expression levels when screening individual transcripts. To perform the normalization, read counts were first summed across all transcripts in each library, and then counts for each transcript were multiplied by the ratio of the largest summed count over the individual library’s summed count. Individual transcripts were then screened by tissue to remove those with no recorded reads or particularly low levels of expression. The latter was identified as any transcript with fewer than 10 normalized counts per library or 100 or fewer total normalized counts. The resulting sets of transcript counts provided a genic profile of the expressed transcripts in liver and gill and also a measure of each transcript’s level of expression. These sets were used to identify the most prevalent transcripts in each tissue, followed by closer comparisons of their expression profiles.

Transcript expressions specific to each tissue, and those common to both tissues were identified. These liver and gill expression profiles were then compared in order to determine whether the tissue specific profiles had in fact been isolated. This was crucial to ensure meaningful comparisons later against the liver and gill transcript libraries from *D. rerio*. For transcripts with shared expression, a Pearson’s correlation coefficient of read counts was calculated between tissues to identify how broadly similar expression was between liver and gill among the set of common active genes. The R package edgeR [22] was used to identify the differentially expressed genes between these tissues using a pairwise comparison and a Benjamin and Hochberg adjusted p-value cutoff of 0.05. The sets of liver specific, gill specific, and differentially expressed shared transcripts were then analyzed for over-represented Gene Ontology terms using the R package GO-seq [23]. Over-represented terms were again identified as those with a Benjamin and Hochberg adjusted p-value of 0.05.

### Comparing gill and liver expression profiles of *P. borchgrevinki* and *D. rerio*

*P. borchgrevinki* liver and gill transcriptomes analyzed above were compared to tissue specific expression profiles from *D. rerio*. Non-normalized zebrafish EST libraries - library 14410 (name: NIH_ZGC_8) for liver, and library 19741 (name: ZF28) for gill, were downloaded from NCBI’s unigene database. Following the same procedures we used for *P. borchgrevinki*, all sequences in the zebrafish EST libraries were assigned functional annotations by performing BLASTx searches with an e-value cutoff of 10^-8^ against SwissProt, followed by UniProt for sequences finding no match in the former. To help reduce the redundancy present in the non-normalized zebrafish EST libraries, we condensed the resulting mappings down to a list of unique UniProt accession numbers, which were then used to identify associated GO terms. A Fisher’s exact test was used to compare the incidence of each GO term shared between the same-tissue transcriptome of the two fish species. Terms were identified as significantly different if Benjamini and Hochberg corrected p-values were 0.05 or below. However, interpretation was approached with caution given the great phylogenetic distance between these species, and differences in sequencing platforms, both of which makes it difficult to discern adaptations to their differing thermal environments. Additionally, while there may have been important differences among species-specific GO terms, we restricted our comparison only to those shared terms as the much shallower sequencing depth of the *D. rerio* libraries meant that many genes and associated GO terms may simply have been missed in this species.

### Comparing liver transcriptomes between notothenioid fishes

The transcriptomes of three notothenioid fishes, *N. coriiceps, P. antarcticum,* and *C. aceratus*, have recently been reported by Shin *et al*. [7]. These include liver specific transcriptomes from specimens collected from their native waters allowing for a direct comparison with the native liver specific slice of the *P. borchgrevinki* reference transcriptome. To compare the genic content between these using OrthoMCL [24], six-frame translations were generated for the cDNA sequences in each species’ transcriptome that were then used to provisionally assign OrthoMCL groupings to the original contigs. The resulting ortholog assignments were compared between transcriptomes to make an estimate of the number of conserved genes.

The transcriptomes were annotated with GO terms as previously described and enriched terms were identified for the set of conserved genes across all four transcriptomes. These genes are of interest as they may include many playing an important role in the cold adaptation and specialization of these fishes. Significance was assessed using a Benjamin and Hochberg adjusted p-value threshold of 0.01 and we retained only those terms associated with at least 10 contigs across the combined transcriptomes. The list of enriched GO terms was then investigated further using the TreeMap in REVIGO [25] to identify major functional classes.

## Results and discussion

The transcriptome, comprised of mRNA from expressed genes, informs on the scope of cellular actions. Understanding the constituents of a transcriptome can therefore be useful in delineating this scope. Additionally, sequence information from either a transcriptome or genome are critically important resources for further analyses into changes in the level of gene expression in native or experimental comparisons using the increasingly adopted approach of RNA-seq. Here, we constructed the first reference transcriptome for a high-Antarctic notothenioid fish, the cryopelagic bald notothen *P. borchgrevinki*. This fish is endemic to the southern-most Antarctic coast (McMurdo Sound, Ross Island), and is the sole notothenioid species to exploit the sub-surface ice platelet layer, ostensibly the coldest and iciest of all Antarctic marine habitats.

The reference transcriptome we constructed in this study, besides providing a resource and model for gene expression the coldest extreme of marine habitats, has the added purpose of enabling our analyses of transcriptional responses of this highly cold-adapted species to thermal stress (to be reported elsewhere). We also hope that it will enable future studies within the Antarctic research community towards understanding the fate of the Antarctic notothenioids as a taxon in face of global temperature change. To this end, the reference transcriptome included source RNA from a broad selection of tissues and temperature conditions (Table 1). These encompass the transcripts expressed in *P. borchgrevinki* at their ambient freezing water temperatures (−1.9°C), during warm acclimation (4°C), and under acute heat stress (rapid warming to its Critical Thermal Maximum). To facilitate community usage of the resource generated in this study, the transcriptome and its provisional annotation have been made available online at http://www.life.illinois.edu/ccheng/pborch/ and the raw read through NCBI’s Sequence Read Archive (Accession SRP018876). In this study, we also used the reference transcriptome to map the abundance of short transcripts obtained by Illumina sequencing of gill and liver of environmental specimens to profile their expression patterns, and compare them to the temperate model the zebrafish, to discern potential cold-specific differences in the types of expressed genes.

### Sequencing and assembly

Roche/454 sequencing of a normalized cDNA library created from the pooled RNA of the tissues listed in Table 1 on one full picotiter plate produced 1,668,712 reads with an average read length of 418 nt, totaling 698,136,301 nt. Ultimately, 1,351,246 of these reads (549,451,576 nt) were aligned using gsAssembler v. 2.6 and assembled into 42,620 contigs. These contigs were divided by gsAssembler into 27,021 isogroups (putative genes) and 35,962 isotigs (putative transcripts). In terms of overall transcriptome size, the 42,620 contigs of *P. borchgrevinki* are comparable to several recently sequenced teleost transcriptomes, namely, 54,921contigs from the guppy *Poecilia reticulata* transcriptome [26], 36,110 contigs from the European eelpout *Zoarces viviparus*[27], and 42,953 contigs from the Pacific herring *Clupea pallasii* transcriptome [28]. It exceeds the number of contigs reported from the assembly of liver transcriptomes in native cold water specimens of the three Antarctic notothenioids sequenced by Shin *et al*. [7]. Our assembly also produced 212,245 singletons which were excluded from the subsequent analysis as their lack of sequencing coverage presented risks of introducing sequencing errors or assembly artifacts into the transcriptome.

**Figure 1:**
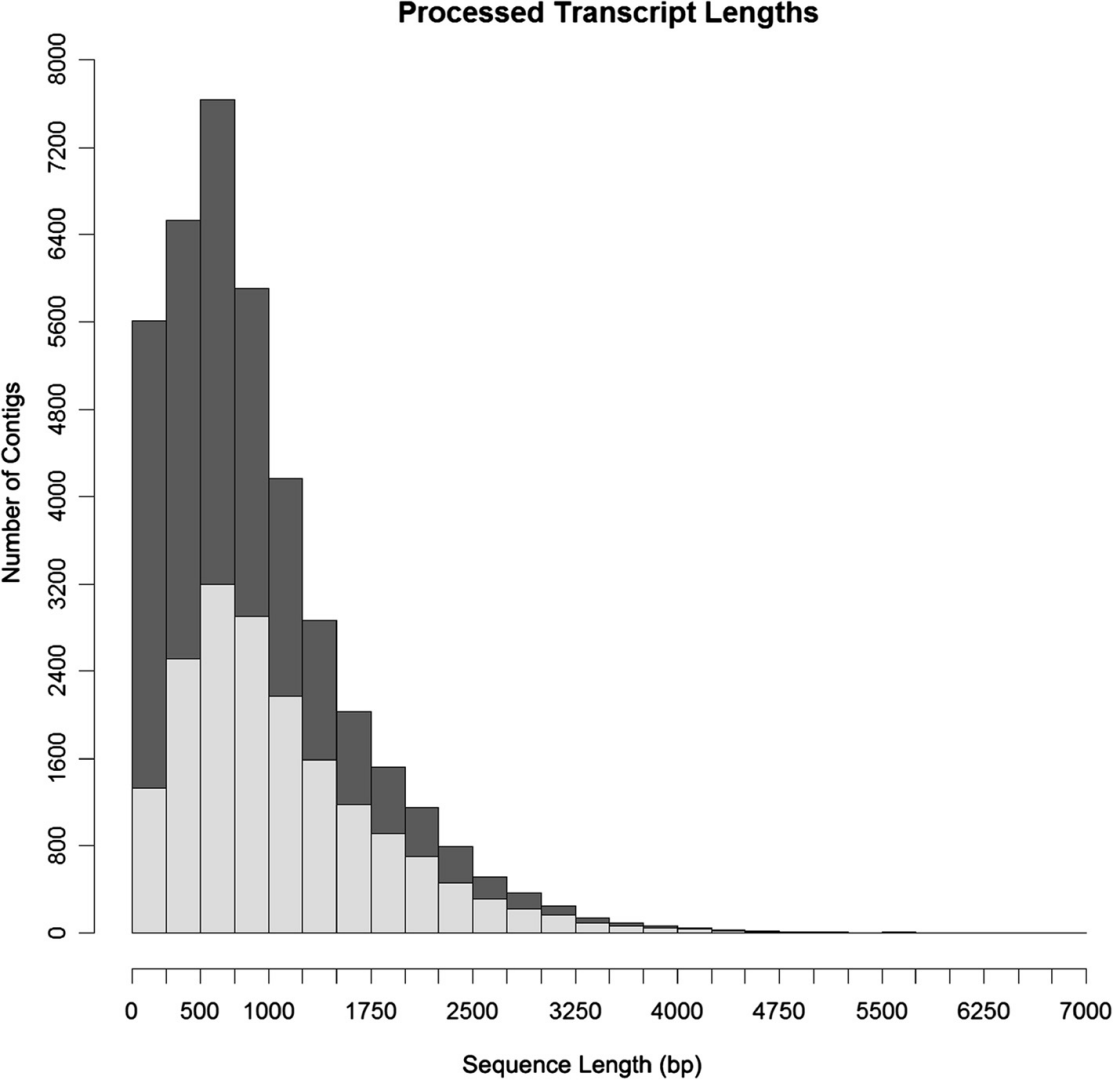
**Contig length distribution in the reference and annotated transcriptomes of *P. borchgrevinki.*** Distributions of contig lengths in the reference transcriptome (dark grey), and the annotated transcriptome (light grey). The former consists of all assembled contigs following clustering while the latter shows only those contigs that could be identified in either SwissProt or UniProt/TrEMBL. The X-axis shows sequence length and the Y-axis the number of contigs that share this length.

We removed 2,558 contigs that are shorter than 100 nt, leaving 40,062 contigs in the provisional reference transcriptome. Clustering to remove near identical sequences condensed 597 other contigs into 293 clusters, leading to a final size of the reference transcriptome of 39,758 contigs. We did not attempt a more aggressive removal of redundancy from the transcriptome as highly similar contigs could be splice variants or closely related members of gene families, both of which are biologically relevant. The transcripts in the reference transcriptome averaged 923±713 nt in length, ranging from 100 to 6826 nt (Figure 1), and with an average read depth of 13.88-fold.

### Annotation of reference transcriptome

The proportions of the reference transcriptome annotated against the four queried databases are shown in Table 2. Screening first against SwissProt followed by UniProt/TrEMBL matched a total of 17,951 contigs, or nearly half (49.7%) of the reference transcriptome, to 13,504 unique hits. These 17,951 contigs comprised many of the longer transcripts in the reference transcriptome (Figure 1), and we categorized this set as the annotated transcriptome. Although searches against the NCBI non-redundant protein and nucleotide databases produced matches for a further 5,909 contigs, these were excluded from the annotated transcriptome as few had available functional annotation.

**Table 2:** **Annotation results of *P. borchgrevinki *reference transcriptome**

**Database**	**Contigs with hits/Total number of sequences**	**Percentage with hits**
**Tiered screening**^**1**^		
SwissProt (BLASTx)	14,889 / 39,758	37.4%
UniProtKB/TrEMBL (BLASTx)	3,062 / 24,869	12.3%
NCBI’s NR (BLASTx)	294 / 21,513	1.3%
NCBI’s NT (BLASTn)	5,615 / 21,219	26.4%
**Screening all sequences**^**2**^		
SwissProt (BLASTx)	14,889 / 39,758	37.4%
UniProtKB/TrEMBL (BLASTx)	17,951 / 39,758	45.1%
NCBI’s NR (BLASTx)	17,877 / 39,758	44.9%
NCBI’s NT (BLASTn)	21,898 / 39,758	55%

Breakdown of the number of contigs with BLAST hits at an e-value of 10^–9^ or lower in each of the screened databases. ^1^The first screening report shows the number of contigs with hits in each database out of those not previously identified. Tiered screening of databases was run in the order of most detailed annotations to least. ^2^The second screening report shows the number of contigs with hits in each database out of the total number of contigs in the reference transcriptome after clustering.

**Figure 2:**
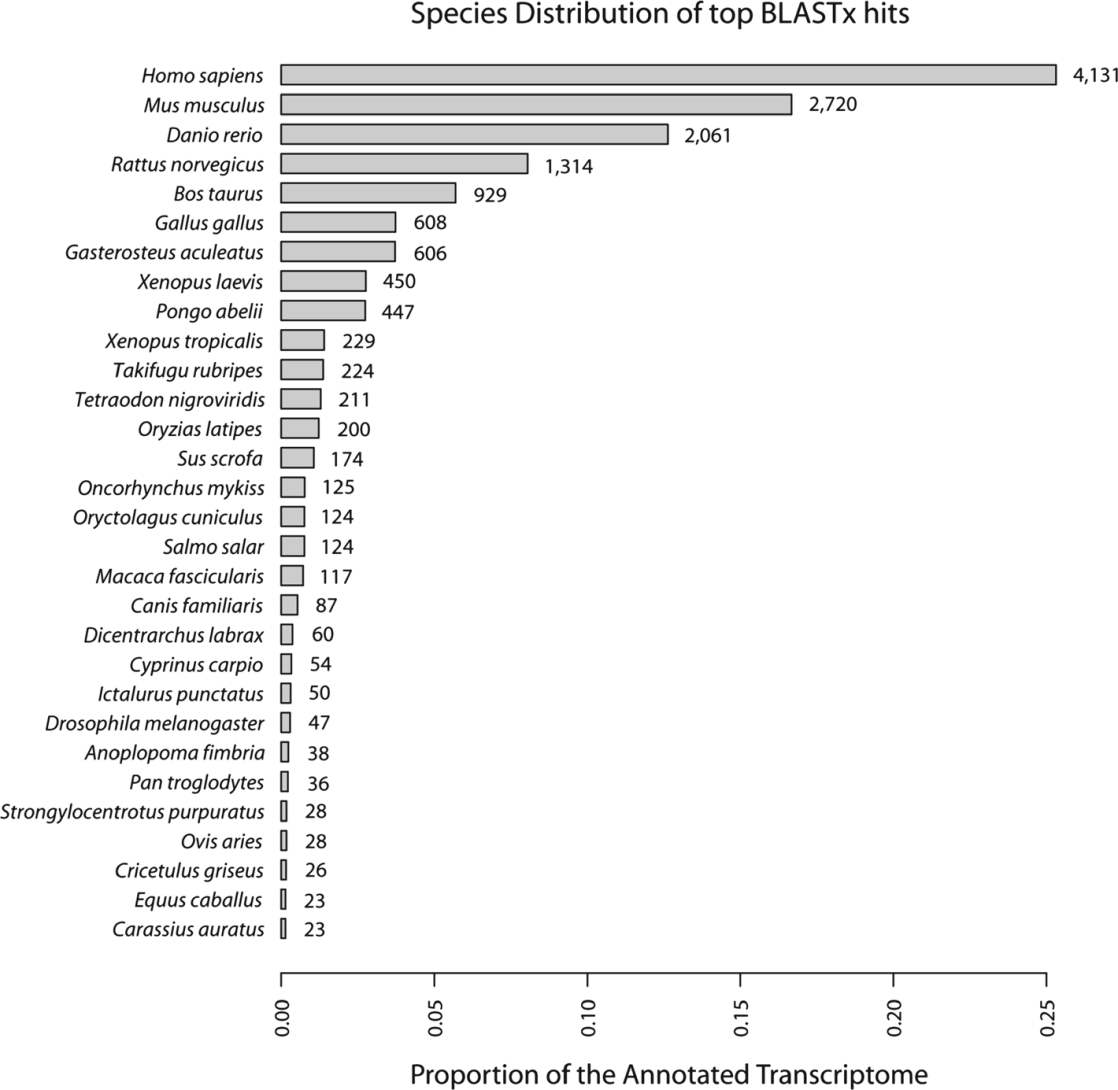
**Species breakdown of the top BLASTx hit for the contigs comprising the annotated transcriptome.** The most prevalent species associated with the top scoring BLASTx hits against SwissProt or Uniprot for the contigs making up the annotated *P. borchgrevinki* transcriptome. Not shown is the breakdown of an additional 1,025 contigs comprising the remainder of the annotated transcriptome that were dispersed over 330 further species. The x-axis shows the proportion of the annotated transcriptome described by each species, with the exact number of associated contigs displayed immediately following each bar.

The taxonomic distribution of the highest scoring BLASTx hits used to identify members of the bald notothen’s annotated transcriptome shows that these largely reflect reported species’ representation within the SwissProt database (Figure 2). There are two notable biases within the annotated transcriptome, the first being against model species, and the second being a general over-representation of fish and amphibians [29].

Among the members of the annotated transcriptome, Gene Ontology (GO) terms could be assigned to 16,057 of the 17,951 contigs (89.5%), collectively describing their many functional roles. The biological process, molecular function, and cellular component ontologies each comprise a number of terms individually defining a discrete role or location. In total, 5,627 distinct terms from the biological process ontology were associated with genes in the annotated transcriptome, 2,507 from the molecular function ontology, and 947 from the cellular component ontology. The terms themselves could be assigned to one or more contigs within the transcriptome, and 167,907 GO assignments were made to the annotated transcriptome with 65,255 assignments from the biological process ontology, 52,468 from the molecular function ontology, and 50,184 from the cellular component ontology.

**Figure 3:**
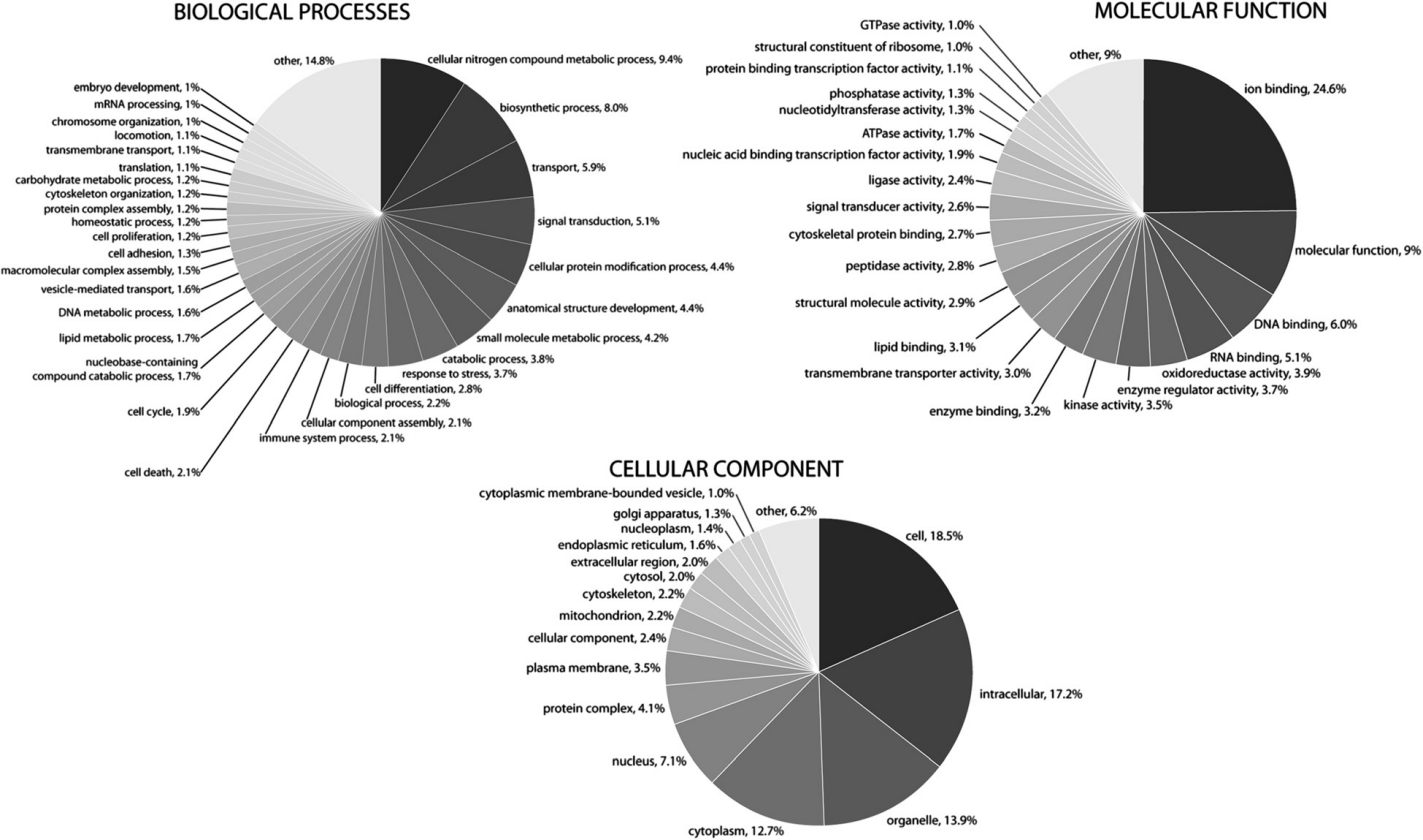
**Breakdown of GO slim terms describing the annotated transcriptome of *P. borchgrevinki.*** The GO assignments for the annotated transcriptome were used to generate a set of GO slim terms. The distribution of these 148 GO slims are graphed in three pie charts showing the frequency of each term in the biological process, molecular function, and cellular component ontologies. Listed percentages show each term’s proportion of total assignments within that ontology with all terms associated with less than 1% of assignments grouped under “Other”.

**Figure 4:**
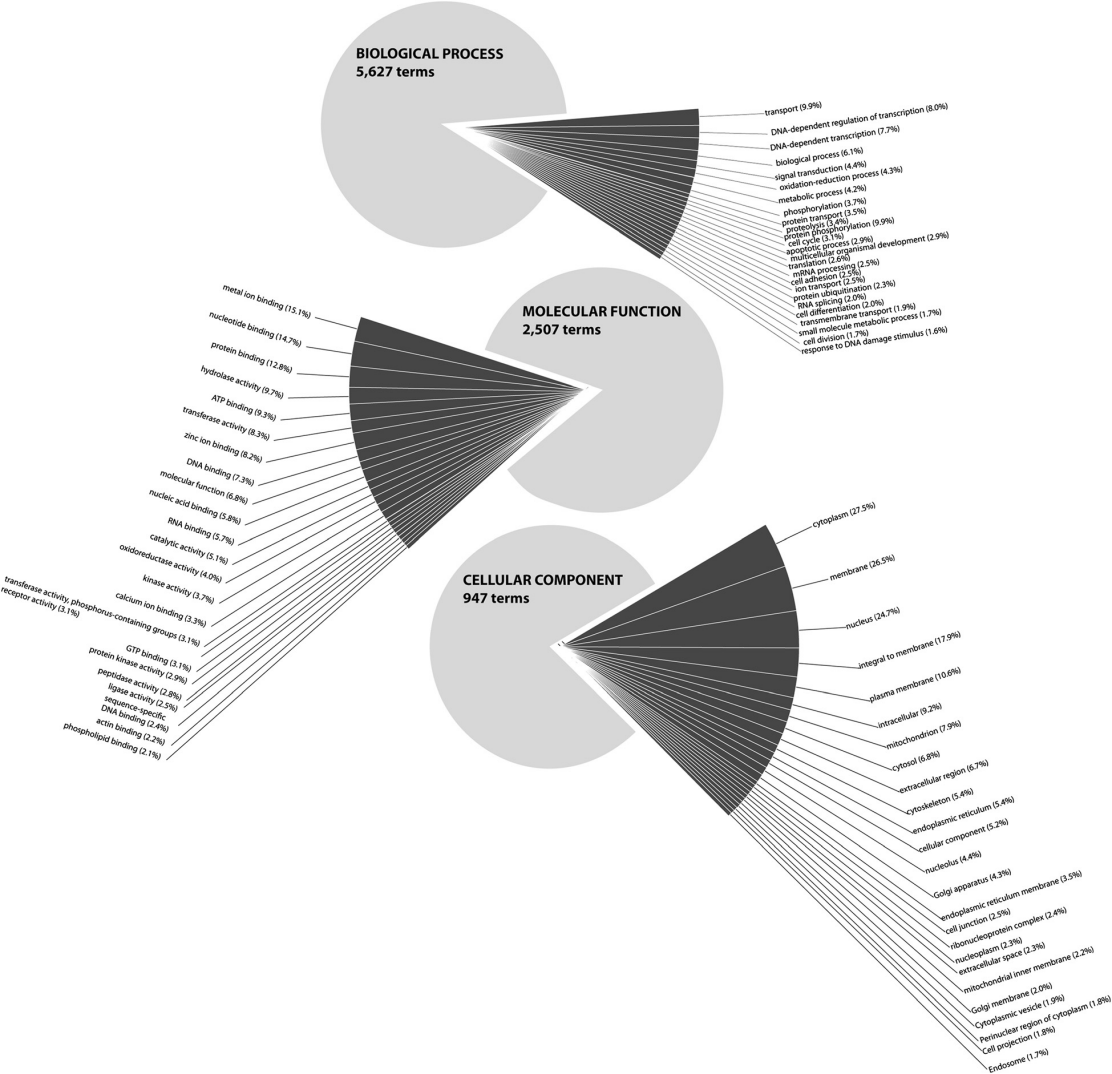
**Breakdown of GO terms associated with members of the annotated transcriptome of *P. borchgrevinki.*** Pie charts showing the prevalence of the 25 most common terms from each ontology in the *P. borchgrevinki* reference transcriptome. Dark grey slices are the proportion of GO assignments from each of the 25 most prevalent terms while the light grey represents the proportion of assignments from all the remaining terms in the ontology. The size of each slice represents the proportion of total GO assignments associated with the term and the listed percentage shows the proportion of the annotated reference transcriptome that the term has been assigned to.

These GO assignments were used to generate a set of GO slim terms that delineate the high-level functional breakdown of the transcriptome (Figure 3). Among the general GO assignments, despite their large number, there is a sharply skewed distribution in how often each term appears. Most terms are associated with relatively few contigs, 18.4 on average, and only a handful of terms are widespread throughout the transcriptome. Only 21 biological process terms, 26 molecular function terms, and 21 cellular component terms were assigned to 2% or more of the contigs in the annotated transcriptome (Figure 4). As illustration, the identities and percent abundance of each ontology’s most prevalent GO terms are presented in Figure 4.

These highly prevalent terms are separately sectioned in the pie chart, while the balance of the chart shows the contributions of all the remaining terms, offering a clear visual depiction that very few terms are widely dispersed throughout the transcriptome. In the Biological Process Ontology, the dominant 25 terms in total account for 9.8% of all assignments, with the remaining 80.2% distributed over the other 5,622 terms. In the molecular function ontology, the dominant terms account for only 15.7% of all assignments, and in the cellular component ontology only 20.0%.

Inspection of the most prevalent GO terms in the biological process ontology suggests the largest contributions to the annotated transcriptome are from genes involved in transport, transcription regulation and protein biosynthesis, cell signaling, as well as those controlling cell proliferation. Compared to the most prevalent biological process terms reported for the *P. crocea* transcriptome [30] the *P. borchgrevinki* transcriptome shows increased representation from oxidation-reduction processes and protein ubiquitination but fewer terms related to metabolism. Looking at the molecular function ontology, the most prevalent terms in the annotated transcriptome primarily relate to binding activity and are similar to those reported from other transcriptomes with notably greater representation from both zinc ion binding and oxidoreductase activity compared to *P. crocea*. As in other reported transcriptomes the cellular component ontology was dominated by the broadest descriptions of location. We next attempted a more quantitative and comprehensive comparison between the transcriptomes of *P. borchgrevinki* and *D. rerio* in order to better identify differences in transcriptome composition.

### Transcriptomic profiling

To assess the completeness of the Antarctic bald notothen annotated transcriptome, we compared it to that of the zebrafish downloaded from Ensmbl [17]. We selected zebrafish for its well delineated transcriptome and for the availability of tissue specific expression profiles. Comparisons across annotated transcriptomes were made on the basis of GO-slim terms, which are a subset of the original GO terms that provide a broad overview of functions, allowing us to identify the high-level transcriptome differences between the two teleost fishes.

**Figure 5:**
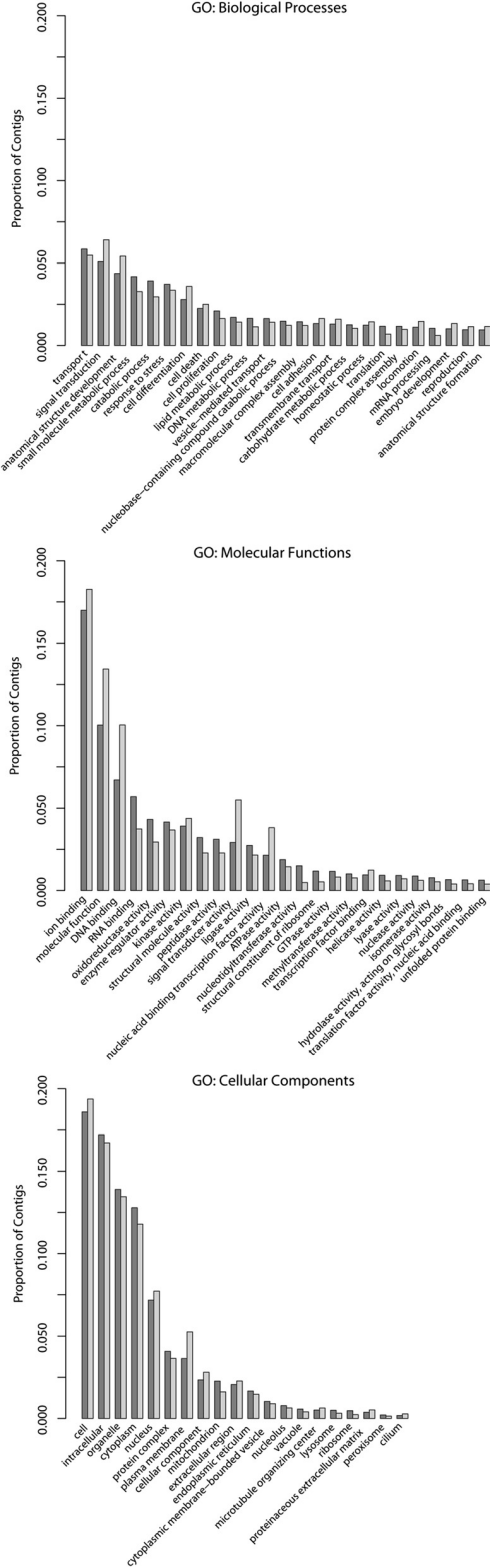
**Comparison of significantly differing GO-slim terms between *P. borchgrevinki *and *D. rerio.*** Where applicable the 25 most prevalent GO-slim terms that significantly differ between bald notothen reference transcriptome and the *D. rerio* transcriptome are presented. The y axis shows the proportion of contigs with that the listed GO-slim term is assigned to, with GO-slim terms identified by name in the x axis. *P. borchgrevinki* values are shown in dark grey and *D. rerio* in light grey.

From the entire GO-slim set of 148 terms, 39 biological process terms, 20 molecular function terms, and 29 cellular component terms were found to significantly differ between annotated transcriptomes suggesting widespread differences. The most prevalent of these are graphed in Figure 5, which shows that the annotated transcriptome is particularly enriched relative to *D. rerio* in terms related to small molecule metabolic processes, transcription and translation, catabolism, as well as oxidoreductase activity, but depauperate relative to *D. rerio* in transcripts associated with signal transduction and development. Of these, the lack of developmental genes quite clearly reflects that only adult bald notothen specimens were used in the constructing the transcriptome, thus genes involved in development would have been transcriptionally silent. The causes of the remaining differences are more difficult to interpret as they could reflect that the tissues used in generating our transcriptome are not all inclusive, the inclusion of transcriptional responses to heat in *P. borchgrevinki*, as well as the disparate evolutionary histories of both species.

### Isolating gill and liver expression profiles

Using the reads from Illumina sequenced liver and gill samples we isolated tissue-specific expression profiles (Table 3). We isolated 13,128 expressed transcripts making up the gill transcriptome, and 10,450 that make up the liver transcriptome. Among these, there was a pronounced difference in the pattern of expression between the two tissues, with liver showing particularly high levels of expression in only a few discrete transcripts while gill expression was more evenly distributed across transcripts.

**Table 3:** Illumina sequencing and mapping results of liver and gill samples

**Tissue (Specimen)**	**No. read**	**Retained reads**	**Annotated transcriptome**			**Reference transcriptome**		
			**% Mapped**	**% Unmapped**	**% Ambiguous**	**% Mapped**	**% Unmapped**	**% Ambiguous**
Liver (1)	47,999,274	31,104,129	68.5	28.0	3.4	75.8	19.8	4.4
Liver (2)	60,009,042	39,254,065	61.9	34.5	3.5	70.9	24.4	4.7
Liver (3)	47,666,040	26,435,958	62.4	33.9	3.6	71.2	24.0	4.8
Gill (1)	71,817,060	42,149,816	50.4	2.1	2.1	64.7	31.6	3.7
Gill (2)	50,157,764	29,490,860	52.9	2.8	44.0	69.7	25.9	4.4
Gill (3)	59,998,042	35,620,006	43.8	1.9	54.2	61.4	34.9	3.7

Results from Illumina sequencing of liver and gill samples from *P. borchgrevinki* held at their natural water temperatures. Sequenced 100 nt reads were processed using the Fastx toolkit and processed to remove the adaptor sequence used in library construction, low quality sections of each read, and any reads of uniformly low quality. Retained reads were thus shorter (80 nt) but of higher quality and used for read mapping with the program Bowtie.

There were notable tissue differences in the transcripts that were present and in the expression of those that were shared. The four most prevalent transcripts in gill were Cytochrome c oxidase subunit 2 (0.94% of all expression), Carbonic anhydrase 1 (0.76%), Elongation factor 1-alpha (0.74%), and Cytochrome c oxidase subunit 1 (0.68%), while in the liver Vitellogenin-1 (6.1% of all expression), Vitellogenin-2 (6.1%), Apolipoprotein A-I (3.4%), and 14kDa-apolipoprotein (3.2%) were most common and far more highly expressed. Based on charting frequencies, a discontinuity is seen in expression following these top four transcripts, with notably reduced levels among the remaining bulk of transcripts. High levels of carbonic anhydrase in gills are well-known as this plays an important role in gas exchange [31], while the other three genes are generally important for their roles in protein biosynthesis and metabolism. In the liver, high levels of expression of the egg yolk precursor protein vitellogenin suggest at least some of the specimens were female, while high levels of expression among apolipoproteins have previously been noted in this tissue in the notothenioid *D. mawsoni*[6].

Comparing the transcripts that were found in the two tissues, 700 were expressed only in the liver and 3,832 only in gill. Of the 9,750 transcripts expressed in both gill and liver, levels of expression were weakly correlated between tissues (Pearson’s correlation coefficient 0.22), and a pairwise comparison using edgeR identified 3,832 of them as significantly differentially expressed. In part to confirm that our approach was successful in separating tissue specific transcription we examined the functional roles of these three sets of transcripts by identifying over-represented gene ontology terms in each.

There were 34 over-represented gene ontology terms associated with the gill-specific transcripts that seem to be primarily defined by changes in receptor activity and signal transduction, particularly G-protein mediated, cell binding, and cell shape (Additional file 1: Table S1). The liver-specific expression corresponded to 124 over-represented terms broadly describing protein synthesis, proteolysis, the innate immune response, redox reactions, along with lipid and cholesterol metabolism, transport, and storage (Additional file 1: Table S2). Looking at those transcripts that were shared but significantly differentially expressed, 29 gene ontology terms were over represented, including additional differences in cellular metabolism and carbohydrate metabolism (Additional file 1: Table S3). These functional differences would seem to suggest that we have been successful in partitioning the members of the reference transcriptome between tissues based on the Illumina read counts.

### Comparing tissue specific transcriptomes between *P. borchgrevinki* and *D. rerio*

Annotation of the 14,293 sequences in the *D. rerio* liver library mapped 13,518 to 2,093 unique putative homologues. Of these, 1,958 had associated GO terms encompassing 15,160 total assignments. For the gill, 5,156 of the 6,352 sequences in the library mapped to 2,278 unique putative homologues, with 2,109 having associated GO terms encompassing 18,258 total assignments. Comparing the liver transcriptomes of the two species, we identified 2,460 GO terms shared between both, 170 of which significantly differed. For the gill transcriptomes, 3,029 terms were shared with 86 significantly differing.

**Figure 6:**
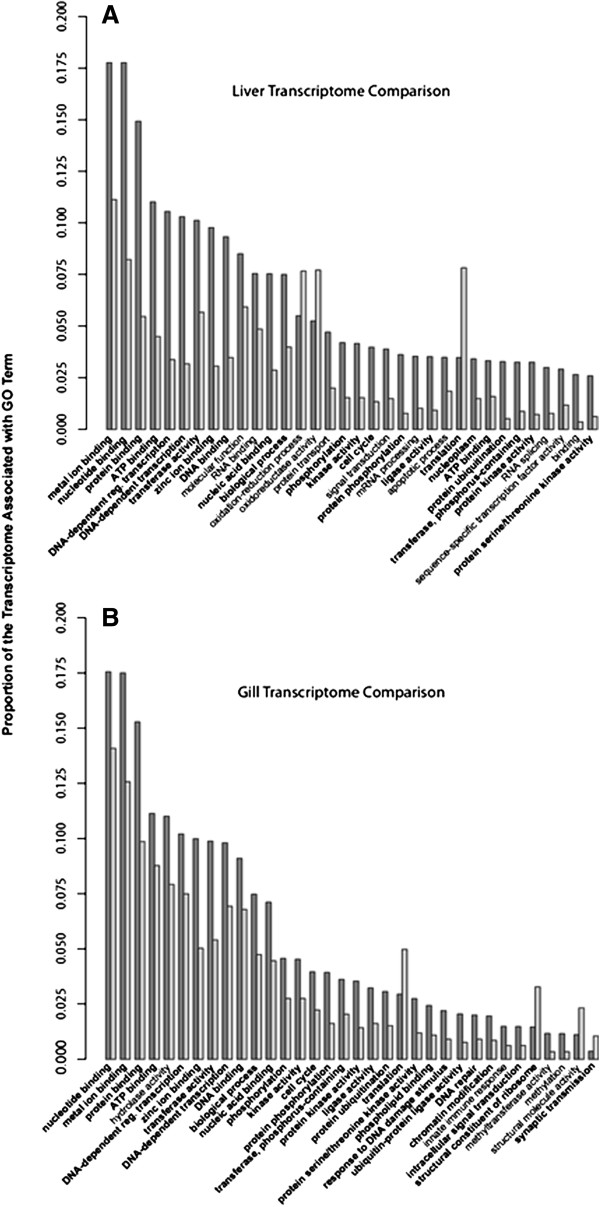
**Differing GO terms between *P. borchgrevinki *and *D. rerio*liver (A) and gill (B) transcriptomes.** Fisher’s exact test was used to compare the incidence of GO terms between tissue specific transcriptomes of *P. borchgrevinki* and *D. rerio*. The comparison of liver transcriptomes is shown in panel **A**, and gill transcriptomes in panel **B**. GO terms were said to significantly differ within tissues where the Benjamini and Hochberg corrected p-value from the two-tailed Fisher’s exact test was 0.05 or lower. The figure shows the 34 most prevalent biological process and molecular function GO terms in *P. borchgrevinki* based on the number contigs each term is associated with. *P. borchgrevinki* is represented by dark grey bars, *D. rerio* by light grey and GO terms that are significantly different in both tissues are shown in bold.

With respect to the significantly differing terms, there was substantial overlap between tissues, with 58 terms shared between gill and liver. This overlapping set is particularly well represented among the most prevalent GO terms (Figure 6). In the more restricted set of heavily represented terms there is an apparent an over-representation of genes related to transcriptional regulation, nucleotide binding, and metal ion binding. Given the great phylogenetic distance between these species caution must be applied in extrapolating biological meaning to these differences. However, several terms among these are of potential interest, as their identifications were corroborated in previous studies, lending to greater confidence of their potential role in cold-adaptation. These include protein binding, which is associated with a number of molecular chaperones, among other proteins, and may suggest that these are more common within the *P. borchgrevinki* transcriptome in addition to being highly expressed at their natural water temperatures [6]. Shared and under-represented relative to *D. rerio* were translation and the ribonucleoprotein complex among biological process and molecular function terms. Tissue-specific differing GO terms included a notable under-representation of genes related to oxidative stress in the liver, while gill showed over-representation in terms related to signal cascades and the cell-cycle.

Shin *et al*. [7] made particular note of increased representation of genes related to the ubiquitination pathway among three notothenioids relative to several temperate and tropical species. Although these were not among the most prevalent significantly different GO terms both protein ubiquitination (GO:0016567) and ubiquitin-protein ligase activity (GO:0004842) were among the broader set of shared over-represented terms in both liver and gill of *P. borchgrevinki* suggesting genes with these functions may similarly be more common in *P. borchgrevinki* as well. Increased representation of these gene functions also corresponds to observations of high levels of protein ubiquitination in these fish at their native freezing water temperatures [10]. Finally, also of note were DNA repair and response to DNA damage, which Buckley and Somero [11] suggested might play an important role in the stress response of polar fishes, were over represented in both tissues (Figure 6), albeit below the threshold to be visible in the figure for the liver.

### Comparing liver transcriptomes between notothenioid fishes

**Figure 7:**
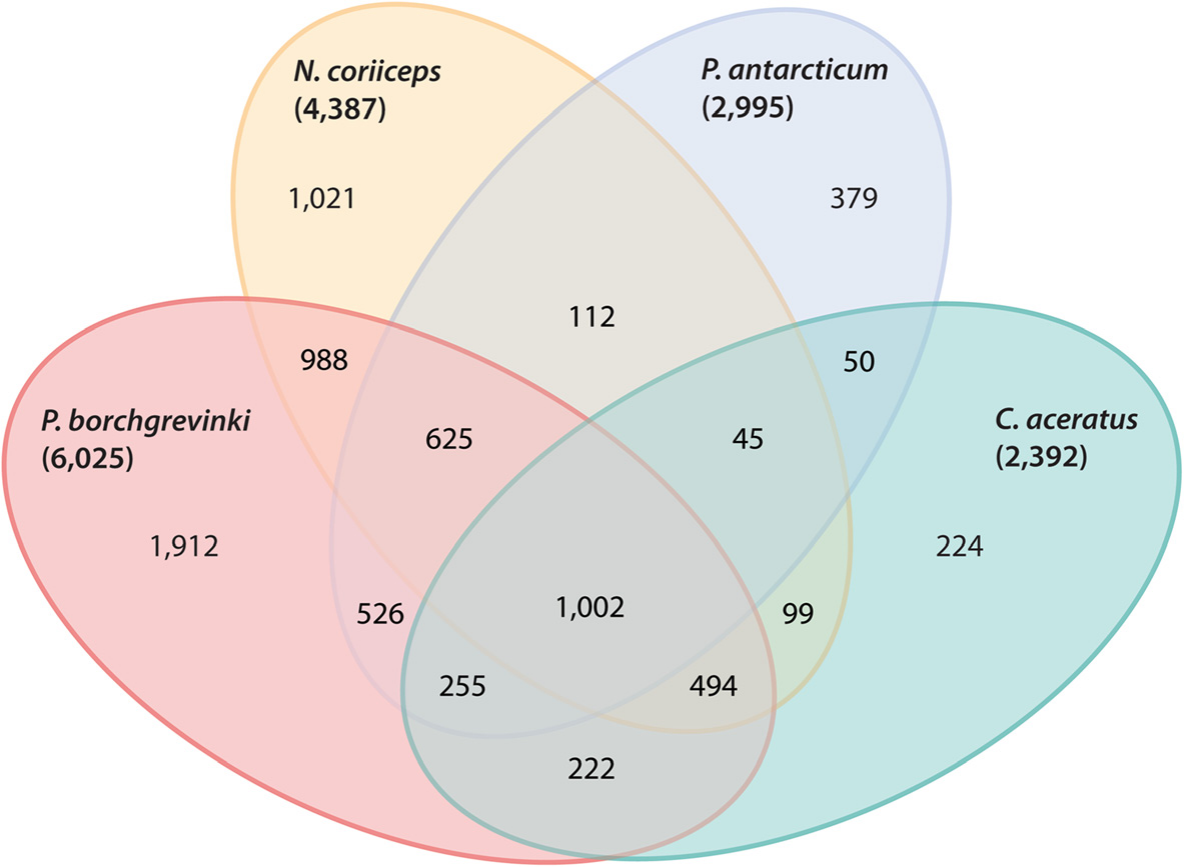
**Comparison between the genic content of notothenioid liver transcriptomes.** Comparison of liver transcriptomes between ambient water specimens of four species of notothenioid fishes. Numbers are from the distinct OrthoMCL assignments made for each transcriptome with the total number shown in parenthesis while overlaps show the number of assignments that are shared between species.

Liver transcriptomes were compared between *P. borchgrevinki* and the three notothenioids sequenced by Shin *et al*. [7]. These species made for an attractive comparison as they were similarly sequenced from a normalized cDNA library and with the same sequencing platform. Comparing these, (Figure 7) the most notable difference seems to come from sequencing depth and success, with far more species specific sequences seen in *P. borchgrevinki* and *N. coriiceps* simply on account of the greater number of contigs for these species. However, there were notable differences among these species and the common set of conserved genes was examined in more detail as these might include many that have played an important role in the cold-adaptation and specialization of the notothenioids.

Compared to the full set of GO terms for all four liver transcriptomes, the conserved set was significantly enriched in 184 terms in the Biological Process ontology, 116 in the Molecular Function ontology, and 65 in the Cellular Component ontology. Within the Biological Process ontology GO terms fell into 14 major categories. Particularly notable among these are groups of GO terms related to ISG15-protein conjugation, response to stress, and a set of GO terms related to fatty acid synthesis all of which have previously been associated with wither acclimation or adaptation to cold. Further groupings included ethanol oxidation, cytokinesis completion of separation, protein heterotrimerization, regulation of protein heterodimerization activity, vesicle-mediated transport, mRNA metabolism, along with five smaller groupings. Within the Molecular Function ontology nine major groupings were found including phospholipid-hydroperoxide glutathione peroxidase activity, ethanolamine kinase activity, translation activator activity, ISG15 ligase activity, GTPase activity, protein disulfide isomerase activity, fatty acid transporter activity, protein binding, and NAD binding. Finally, the Cellular Component ontology included three major groupings namely polysome, melanosome, and the respiratory chain, which are likely related to the role of the liver rather than any shared cold-modifications to these transcriptomes.

That these functional roles are found among the shared set of genes may suggest that some play a role in the cold-adaptation and specialization of these fishes. However, a fully comprehensive comparison must wait until more notothenioid transcriptomes become available including those of the basal temperate lineages.

## Conclusion

We constructed a reference transcriptome for the high-latitude cryopelagic Antarctic nototheniid fish *P. borchgrevinki*, representing a model of gene expression in extreme cold. Using source material that spanned multiple tissues from environmental fish, and chronically and acutely heat stressed fish, this transcriptome was built with the added intention of serving as community resource to facilitate investigations into the molecular basis of evolutionary cold adaptations as well as thermal plasticity of the cold stenothermal notothenioid species in today’s changing climate. Assembly of the Roche 454 sequenced reads from the normalized library resulted in over 40,000 contigs, with putative homologous identified for 17,951 of them. Based on GO-slim functional annotations, this annotated portion of the *P. borchgrevinki* transcriptome showed widespread differences from the transcriptome of the temperate model fish species *D. rerio*. To test the utility of this reference transcriptome for RNA-seq experiments, we mapped short Illumina reads from the gill and liver of cold acclimatized specimens. These mappings allowed us to partition tissue specific expression profiles from fish in their natural freezing waters and compare them to similar profiles available for zebrafish. From this the two *P. borchgrevinki* tissue transcriptomes were both enriched in GO terms for transcriptional regulation, protein binding including putative molecular chaperones, and protein ubiquitination. This suggests that genes with these functions are more prevalent in this Antarctic notothenioid and they may play an important role in this group’s cold adaptation. Currently, we are in the process of using this to investigate the transcriptional changes that occur in this fish over the first four days of exposure to 4°C in order to better understand how their response to heat has been influenced by the long evolution of Antarctic notothenioid fishes in constant freezing water temperatures.

## Competing interests

The authors declare that they have no competing interests.

## Authors’ contributions

KTB and C-HCC were responsible for collection of tissue samples. KTB performed RNA extractions and preparation, sequence assembly, annotation, analysis. C-HCC developed the project design and provided project oversight. Both KTB and C-HCC contributed to the writing of the manuscript. All authors read and approved the final manuscript.

## Supplementary Material

Additional file 1: Table S1. Over represented GO terms in liver specific genes. **Table S2**, Over represented GO terms in Gill specific genes. **Table S3**, Over represented GO terms among differentially expressed but shared genes in both liver and gill. Click here for file
