# Supplementary material for: Model of gene expression in extreme cold - reference transcriptome for the high-Antarctic cryopelagic notothenioid fish Pagothenia borchgrevinki
Source: BMC Genomics. 2013 Sep 21;14:634. doi: 10.1186/1471-2164-14-634 (PMC3856495; doi:10.1186/1471-2164-14-634)
Supplement: Additional file 1: Table S1. — Over represented GO terms in liver specific genes. Table S2, Over represented GO terms in Gill specific genes. Table S3, Over represented GO terms among differentially expressed but shared genes in both liver and gill. [file 1471-2164-14-634-S1.docx]

**Supplimentary Table Legends**

**Supplementary Table 1. Over represented GO terms in liver specific genes**

**Supplementary Table 2. Over represented GO terms in Gill specific genes**

**Supplementary Table 3. Over represented GO terms among differentially expressed but shared genes in both liver and gill.**

**Supplementary Table 1, enriched GO terms in liver specific transcripts**

| **GO term** | **Description** | **no. in tissue** | **no. in reference** |
| --- | --- | --- | --- |
|  |  |  |  |
| GO:0016705 | oxidoreductase activity, acting on paired donors, with incorporation or reduction of molecular oxygen | 28 | 91 |
| GO:0006629 | lipid metabolic process | 27 | 279 |
| GO:0006956 | complement activation | 24 | 54 |
| GO:0005777 | peroxisome | 23 | 130 |
| GO:0070330 | aromatase activity | 21 | 37 |
| GO:0008202 | steroid metabolic process | 17 | 52 |
| GO:0008201 | heparin binding | 15 | 83 |
| GO:0019835 | cytolysis | 15 | 73 |
| GO:0008203 | cholesterol metabolic process | 15 | 46 |
| GO:0042632 | cholesterol homeostasis | 15 | 36 |
| GO:0006953 | acute-phase response | 14 | 35 |
| GO:0005044 | scavenger receptor activity | 13 | 47 |
| GO:0006869 | lipid transport | 12 | 77 |
| GO:0005579 | membrane attack complex | 11 | 14 |
| GO:0016597 | amino acid binding | 10 | 21 |
| GO:0004869 | cysteine-type endopeptidase inhibitor activity | 10 | 32 |
| GO:0006144 | purine base metabolic process | 9 | 28 |
| GO:0016712 | oxidoreductase activity, acting on paired donors | 9 | 18 |
| GO:0042730 | fibrinolysis | 9 | 17 |
| GO:0030212 | hyaluronan metabolic process | 9 | 16 |
| GO:0006547 | histidine metabolic process | 9 | 11 |
| GO:0005319 | lipid transporter activity | 8 | 10 |
| GO:0005577 | fibrinogen complex | 8 | 10 |
| GO:0006641 | triglyceride metabolic process | 7 | 21 |
| GO:0048844 | artery morphogenesis | 7 | 12 |
| GO:0042157 | lipoprotein metabolic process | 7 | 17 |
|  |  |  |  |

| **GO term** | **Description** | **no. in tissue** | **no. in reference** |
| --- | --- | --- | --- |
|  |  |  |  |
| GO:0005576 | extracellular region | 234 | 1210 |
| GO:0005615 | extracellular space | 125 | 419 |
| GO:0016787 | hydrolase activity | 109 | 1747 |
| GO:0003824 | catalytic activity | 101 | 923 |
| GO:0055114 | oxidation-reduction process | 73 | 785 |
| GO:0016491 | oxidoreductase activity | 73 | 735 |
| GO:0006508 | proteolysis | 66 | 626 |
| GO:0045087 | innate immune response | 62 | 281 |
| GO:0010951 | negative regulation of endopeptidase activity | 58 | 86 |
| GO:0008233 | peptidase activity | 57 | 517 |
| GO:0004866 | endopeptidase inhibitor activity | 49 | 65 |
| GO:0006958 | complement activation, classical pathway | 49 | 89 |
| GO:0004252 | serine-type endopeptidase activity | 49 | 169 |
| GO:0006957 | complement activation, alternative pathway | 46 | 58 |
| GO:0008236 | serine-type peptidase activity | 44 | 159 |
| GO:0005789 | endoplasmic reticulum membrane | 43 | 629 |
| GO:0007596 | blood coagulation | 42 | 210 |
| GO:0004867 | serine-type endopeptidase inhibitor activity | 42 | 92 |
| GO:0005792 | microsome | 40 | 233 |
| GO:0005506 | iron ion binding | 38 | 191 |
| GO:0006954 | inflammatory response | 38 | 172 |
| GO:0030414 | peptidase inhibitor activity | 35 | 99 |
| GO:0010466 | negative regulation of peptidase activity | 35 | 100 |
| GO:0007599 | hemostasis | 34 | 66 |
| GO:0004497 | monooxygenase activity | 32 | 85 |
| GO:0020037 | heme binding | 30 | 150 |
| GO:0005102 | receptor binding | 29 | 165 |
| GO:0009055 | electron carrier activity | 28 | 178 |
|  |  |  |  |

**Supplementary Table 1, enriched GO terms in liver specific transcripts (continued)**

| **GO term** | **Description** | **no. in tissue** | **no. in reference** |
| --- | --- | --- | --- |
|  |  |  |  |
| GO:0009116 | nucleoside metabolic process | 6 | 20 |
| GO:0030162 | regulation of proteolysis | 6 | 19 |
| GO:0043499 | eukaryotic cell surface binding | 6 | 18 |
| GO:0007338 | single fertilization | 6 | 17 |
| GO:0030301 | cholesterol transport | 6 | 15 |
| GO:0034362 | low-density lipoprotein particle | 6 | 14 |
| GO:0043498 | cell surface binding | 6 | 11 |
| GO:0018298 | protein-chromophore linkage | 6 | 13 |
| GO:0016763 | transferase activity, transferring pentosyl groups | 5 | 14 |
|  |  |  |  |
|  |  |  |  |
|  |  |  |  |
|  |  |  |  |
|  |  |  |  |
|  |  |  |  |
|  |  |  |  |
|  |  |  |  |
|  |  |  |  |
|  |  |  |  |
|  |  |  |  |

| **GO term** | **Description** | **no. in tissue** | **no. in reference** |
| --- | --- | --- | --- |
|  |  |  |  |
| GO:0016811 | hydrolase activity, acting on carbon-nitrogen bonds | 5 | 13 |
| GO:0035999 | tetrahydrofolate interconversion | 5 | 12 |
| GO:0015695 | organic cation transport | 5 | 11 |
| GO:0034361 | very-low-density lipoprotein particle | 5 | 11 |
| GO:0017127 | cholesterol transporter activity | 5 | 10 |
| GO:0070328 | triglyceride homeostasis | 5 | 10 |
| GO:0070328 | triglyceride homeostasis | 5 | 10 |
|  |  |  |  |
|  |  |  |  |
|  |  |  |  |
|  |  |  |  |
|  |  |  |  |
|  |  |  |  |
|  |  |  |  |
|  |  |  |  |
|  |  |  |  |
|  |  |  |  |
|  |  |  |  |

**Supplementary Table 2, enriched GO terms in gill specific transcripts**

| **GO term** | **Description** | **no. in tissue** | **no. in reference** |
| --- | --- | --- | --- |
|  |  |  |  |
| GO:0016021 | integral to membrane | 672 | 3,214 |
| GO:0005886 | plasma membrane | 488 | 1,913 |
| GO:0005576 | extracellular region | 337 | 1,210 |
| GO:0007165 | signal transduction | 250 | 804 |
| GO:0005856 | cytoskeleton | 247 | 978 |
| GO:0004872 | receptor activity | 187 | 565 |
| GO:0007155 | cell adhesion | 154 | 451 |
| GO:0005543 | Phospholipid binding | 126 | 379 |
| GO:0003779 | actin binding | 110 | 410 |
| GO:0004871 | signal transducer activity | 94 | 291 |
| GO:0005887 | integral to plasma membrane | 93 | 296 |
| GO:0030246 | carbohydrate binding | 92 | 269 |
| GO:0007186 | G-protein coupled receptor protein signaling pathway | 80 | 209 |
| GO:0042995 | cell projection | 97 | 333 |
| GO:0004930 | G-protein coupled receptor activity | 60 | 139 |
| GO:0005578 | extracellular matrix | 70 | 188 |
| GO:0006955 | immune response | 69 | 187 |
| GO:0043547 | positive regulation of GTPase activity | 63 | 193 |
|  |  |  |  |
|  |  |  |  |
|  |  |  |  |
|  |  |  |  |
|  |  |  |  |
|  |  |  |  |

| **GO term** | **Description** | **no. in tissue** | **no. in reference** |
| --- | --- | --- | --- |
|  |  |  |  |
| GO:0005096 | GTPase activator activity | 61 | 186 |
| GO:0005085 | guanyl-nucleotide exchange factor activity | 48 | 147 |
| GO:0031012 | extracellular matrix | 40 | 160 |
| GO:0005089 | Rho guanyl-nucleotide exchange factor activity | 37 | 87 |
| GO:0035023 | regulation of Rho protein signal transduction | 35 | 84 |
| GO:0009897 | external side of plasma membrane | 31 | 78 |
| GO:0030030 | cell projection organization | 30 | 70 |
| GO:0005604 | basement membrane | 25 | 58 |
| GO:0003823 | antigen binding | 23 | 41 |
| GO:0070098 | chemokine-mediated signaling pathway | 15 | 24 |
| GO:0004950 | chemokine receptor activity | 15 | 21 |
| GO:0007257 | activation of JUN kinase activity | 13 | 21 |
| GO:0030890 | positive regulation of B cell proliferation | 13 | 19 |
| GO:0031224 | intrinsic to membrane | 10 | 14 |
|  |  |  |  |
|  |  |  |  |
|  |  |  |  |
|  |  |  |  |
|  |  |  |  |
|  |  |  |  |
|  |  |  |  |

**Supplementary Table 3, enriched GO terms among differentially expressed genes in *P. borchgrevinki* liver and gill samples**

| **GO term** | **Description** | **no. DE** | **no. in reference** |
| --- | --- | --- | --- |
|  |  |  |  |
| GO:0006006 | glucose metabolic process | 33 | 67 |
| GO:0006635 | fatty acid beta-oxidation | 31 | 50 |
| GO:0016616 | oxidoreductase activity, acting on the CH-OH group of donors, NAD or NADP as acceptor | 30 | 54 |
| GO:0018279 | protein N-linked glycosylation via asparagine | 23 | 35 |
| GO:0016747 | transferase activity, transferring acyl groups other than amino-acyl groups | 18 | 27 |
| GO:0006164 | purine nucleotide biosynthetic process | 17 | 31 |
| GO:0003995 | acyl-CoA dehydrogenase activity | 15 | 20 |
| GO:0019825 | oxygen binding | 15 | 25 |
| GO:0005782 | peroxisomal matrix | 14 | 19 |
| GO:0005833 | hemoglobin complex | 13 | 20 |
| GO:0005344 | oxygen transporter activity | 13 | 22 |
| GO:0015671 | oxygen transport | 13 | 22 |
|  |  |  |  |
|  |  |  |  |
|  |  |  |  |
|  |  |  |  |

| **GO term** | **Description** | **no. DE** | **no. in reference** |
| --- | --- | --- | --- |
|  |  |  |  |
| GO:0016740 | transferase activity | 412 | 1490 |
| GO:0005739 | mitochondrion | 390 | 1431 |
| GO:0005783 | endoplasmic reticulum | 297 | 972 |
| GO:0003824 | catalytic activity | 282 | 923 |
| GO:0008152 | metabolic process | 271 | 765 |
| GO:0055114 | oxidation-reduction process | 243 | 785 |
| GO:0016491 | oxidoreductase activity | 235 | 735 |
| GO:0005789 | endoplasmic reticulum membrane | 198 | 629 |
| GO:0044281 | small molecule metabolic process | 119 | 322 |
| GO:0006629 | lipid metabolic process | 111 | 279 |
| GO:0005975 | carbohydrate metabolic process | 92 | 269 |
| GO:0005777 | peroxisome | 63 | 130 |
| GO:0016746 | transferase activity, transferring acyl groups | 56 | 148 |
| GO:0006631 | fatty acid metabolic process | 49 | 92 |
| GO:0044255 | cellular lipid metabolic process | 36 | 61 |
| GO:0050660 | flavin adenine dinucleotide binding | 35 | 70 |
|  |  |  |  |
|  |  |  |  |
|  |  |  |  |
|  |  |  |  |
